# Solvated Electron‐Driven Stepwise ORR on Na‐Metalated N‐Rich Carbon Nitride for Efficient Photocatalytic H_2_O_2_ Production

**DOI:** 10.1002/advs.202516471

**Published:** 2025-10-20

**Authors:** Jianan Feng, Weilin Qin, Li Shangguan, Hui Zhang, Jianhua Sun, Shunping Sun, Yu Guo, Weiwei Lei

**Affiliations:** ^1^ School of Chemistry and Chemical Engineering Institute of Advanced Functional Materials for Energy Jiangsu University of Technology Changzhou 213001 China; ^2^ School of Materials Engineering Jiangsu University of Technology Changzhou 213001 China; ^3^ State Key Laboratory of Materials‐Oriented Chemical Engineering College of Chemistry and Chemical Engineering Nanjing Tech University Nanjing 211816 China; ^4^ School of Science RMIT University Melbourne VIC 3000 Australia

**Keywords:** Na‐metalated, N‐rich, photocatalytic H_2_O_2_ production, polymetric carbon nitride, solvated electrons

## Abstract

Solvated electrons (SEs), known for their strong reductive capability, are widely utilized in diverse catalytic reactions. However, their application in photocatalytic oxygen reduction reactions (ORR) for hydrogen peroxide (H_2_O_2_) production remains largely unexplored. In this work, a Na‐metalated, N‐rich poly(heptazine imide)‐type carbon nitride (PH‐C_3_N_5_‐Na), synthesized via a NaCl/LiCl molten salt‐assisted method is reported, as a novel photocatalyst for in situ SE‐driven H_2_O_2_ production. The N‐rich units and Na into Polymetric carbon nitride (PCN) synergistically narrows the bandgap and facilitates rapid electron transfer, thereby enhancing visible‐light absorption and promoting efficient charge separation. Notably, Na metalation enables the generation of solvated electrons under light irradiation, which mediates a stepwise 2e^−^ ORR pathway via superoxide radicals (•O_2_^−^), significantly boosting H_2_O_2_ production. As a result, PH‐C_3_N_5_‐Na delivers an impressive H_2_O_2_ production rate of 245.51 µmol·h^−1^, 206 times higher than that of PM‐C_3_N_5_‐550, accompanied by an exceptional apparent quantum efficiency of 54.4% (AQE) at 420 nm. This study presents a new strategy for the rational design of high‐performance photocatalysts and unveils the critical role of solvated electrons in enhancing photocatalytic ORR for sustainable H_2_O_2_ synthesis.

## Graphical abstract

A sodium‐metalated, nitrogen‐rich carbon nitride (PH‐C_3_N_5_‐Na) is developed to generate solvated electrons (SEs) under visible‐light irradiation via a eutectic NaCl/LiCl molten salt‐assisted method. The in situ generated SEs drive a stepwise oxygen reduction via •O_2_^−^. Thus, an exceptional quantum efficiency of 54.4% (420 nm) is achieved.

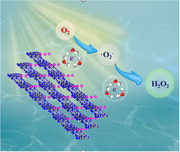

## 1 Introduction

Hydrogen peroxide (H_2_O_2_) is an essential and versatile chemical with broad applications across energy conversion, environmental remediation, and various industrial processes.^[^ ^1^, ^2^, ^3^ ^]^ Compared to the traditional anthraquinone oxidation process, the photocatalytic oxygen reduction reaction (ORR) offers a green and sustainable alternative for H_2_O_2_ production.^[^ ^3^, ^4^, ^5^ ^]^ However, the development of efficient and economically viable photocatalysts remains a key challenge and a major focus of ongoing research.^[^ ^6^ ^]^

It is well known that the superoxide radical (•O_2_^−^) is a key intermediate in the photocatalytic ORR for H_2_O_2_ production.^[^ ^7^, ^8^ ^]^ However, the photocatalytic efficiency is often hindered by the limited formation of •O_2_^−^.^[^ ^4^ ^]^ In this regard, the development of novel reducing agents capable of enhancing the generation rate of •O_2_^−^ is crucial for improving the photocatalytic efficiency of H_2_O_2_ production.

Solvated electrons (SEs), a novel class of reducible species stabilized in solvents, have garnered worldwide interest within the domain of pollutant degradation and green organic synthesis.^[^ ^9^, ^10^, ^11^, ^12^ ^]^ Given its standard reduction potential of −2.9 V, SEs are expected to efficiently reduce O_2_, leading to the formation of •O_2_^−^.^[^ ^12^, ^13^, ^14^ ^]^ In this respect, the exploration of photocatalysts capable of in situ generating solvated electrons under visible‐light irradiation is particularly promising for enhancing the efficiency of photocatalytic ORR toward H_2_O_2_ production.

Polymetric carbon nitride (PCN), a fascinating organic visible‐light photocatalysts, has emerged as a promising candidate for photocatalytic H_2_O_2_ production owning its merits such as appropriated band structure, superior stability, and tunability of chemical structure.^[^ ^15^, ^16^ ^]^ More interestingly, owning to its distinctive “nitrogen cavities” containing six nitrogen lone‐electron pairs, polymeric carbon nitride (PCN) is an outstanding candidate for supporting alkali metals, leading to the formation of alkali‐metalated carbon nitride.^[^ ^17^, ^18^, ^19^ ^]^ Recent studies have demonstrated that PCN with alkali metal can in situ generate solvated electrons under visible‐light irradiation, thereby enhancing the reduction potential and accelerating the transfer rate of photoexcited electrons. For instance, Ou and coworkers reported that potassium heptazine‐based melon polymer (PC‐HM) can produce solvated electrons under visible light, enabling H_2_O_2_ production via oxygen reduction.^[^ ^20^ ^]^ Our group previously developed a fully condensed sulfur‐doped poly(heptazine imide) metalated with sodium (Na‐SPHI), which enabled the in situ generation of solvated electrons and achieved an ultra‐high apparent quantum efficiency for photocatalytic hydrogen evolution.^[^ ^21^ ^]^ These findings suggest that alkali Metalation of carbon nitride is a critical prerequisite for the in situ photocatalytic generation of solvated electrons.

Recently, C_3_N_5_, a novel N‐rich polymeric carbon nitride, has garnered increasing interest due to its low band gap (2.2 eV) and enhanced electron transfer, both of which contribute to improved visible light photocatalytic performance.^[^ ^22^, ^23^, ^24^ ^]^ Notably, C_3_N_5_ contains more electron‐rich N atoms, which not only provides abundant coordination sites for confining metal atoms but also enhances electron delocalization within the conjugated system.^[^ ^22^, ^25^ ^]^ Therefore, it can be anticipated that C_3_N_5_, due to its higher nitrogen content and stronger coordination capability, is more amenable to metalated with alkali metals, thereby enabling the in situ generation of solvated electrons under visible‐light irradiation. However, to date, there have been no reports on the alkali metalation of C_3_N_5_ specifically for the photocatalytic generation of solvated electrons toward efficient H_2_O_2_ production.

Inspired by these insights, a Na‐metalated, N‐rich poly(heptazine imide)‐type carbon nitride (PH‐C_3_N_5_‐Na) was rationally designed to enable the in situ generation of solvated electrons for driving the photocatalytic ORR toward efficient H_2_O_2_ production. The resulting PH‐C_3_N_5_‐Na not only broadens visible‐light absorption and enhances the separation of photogenerated charge carriers, but also facilitates the generation of solvated electrons that effectively promote the formation of superoxide radicals (•O_2_^−^), a key intermediate in the ORR pathway. Leveraging these synergistic effects, PH‐C_3_N_5_‐Na exhibits a markedly enhanced photocatalytic H_2_O_2_ production rate of 245.51 µmol·h^−1^, coupled with an unprecedented apparent quantum efficiency of 54.4% under 420 nm irradiation. In addition, the stepwise 2e^−^ ORR mechanism underlying photocatalytic H_2_O_2_ production was systematically elucidated.

## 2 Results and Discussion

### 2.1 Synthesis and Characterization of PH‐C_3_N_5_‐Na

**Figure 1:**
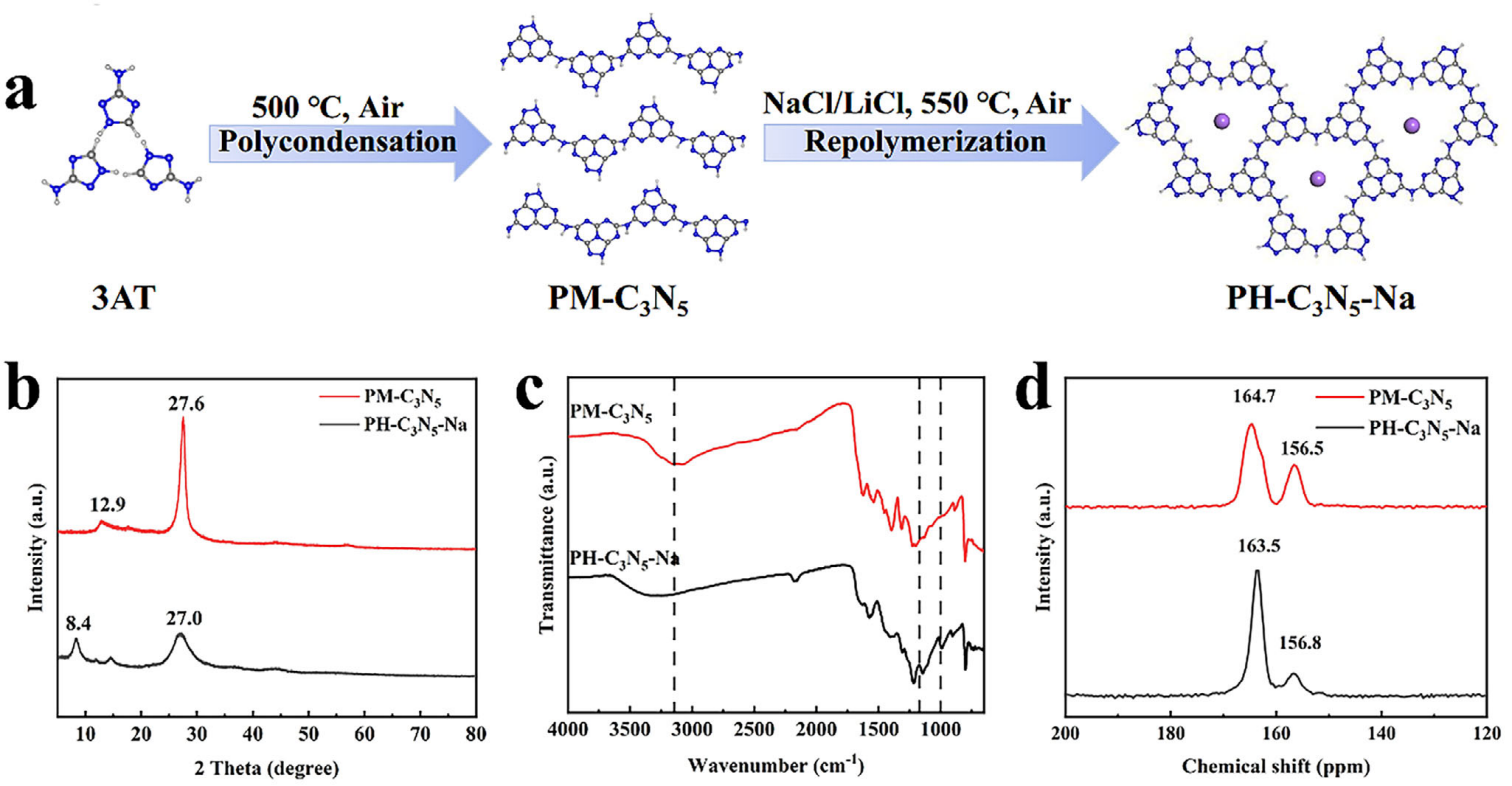
Schematic depiction of the synthetic process for PH‐C_3_N_5_‐Na a), XRD patterns b), FTIR spectra c), and ^13^C NMR spectra d) of PM‐C_3_N_5_ and PH‐C_3_N_5_‐Na.

PH‐C_3_N_5_‐Na was synthesized via a two‐step molten salt‐mediated approach, as depicted in **Figure** **1**a. First, pristine N‐rich melon‐type carbon nitride (PM‐C_3_N_5_) was obtained by thermal condensation of 3‐amino‐1,2,4‐triazole. Subsequently, as‐prepared PM‐C_3_N_5_ was mixed with a eutectic NaCl/LiCl salt and subjected to a post‐polymerization treatment, affording Na‐metalated N‐rich poly(heptazine imide)‐type carbon nitride, denoted as PH‐C_3_N_5_‐Na.

**Figure 2:**
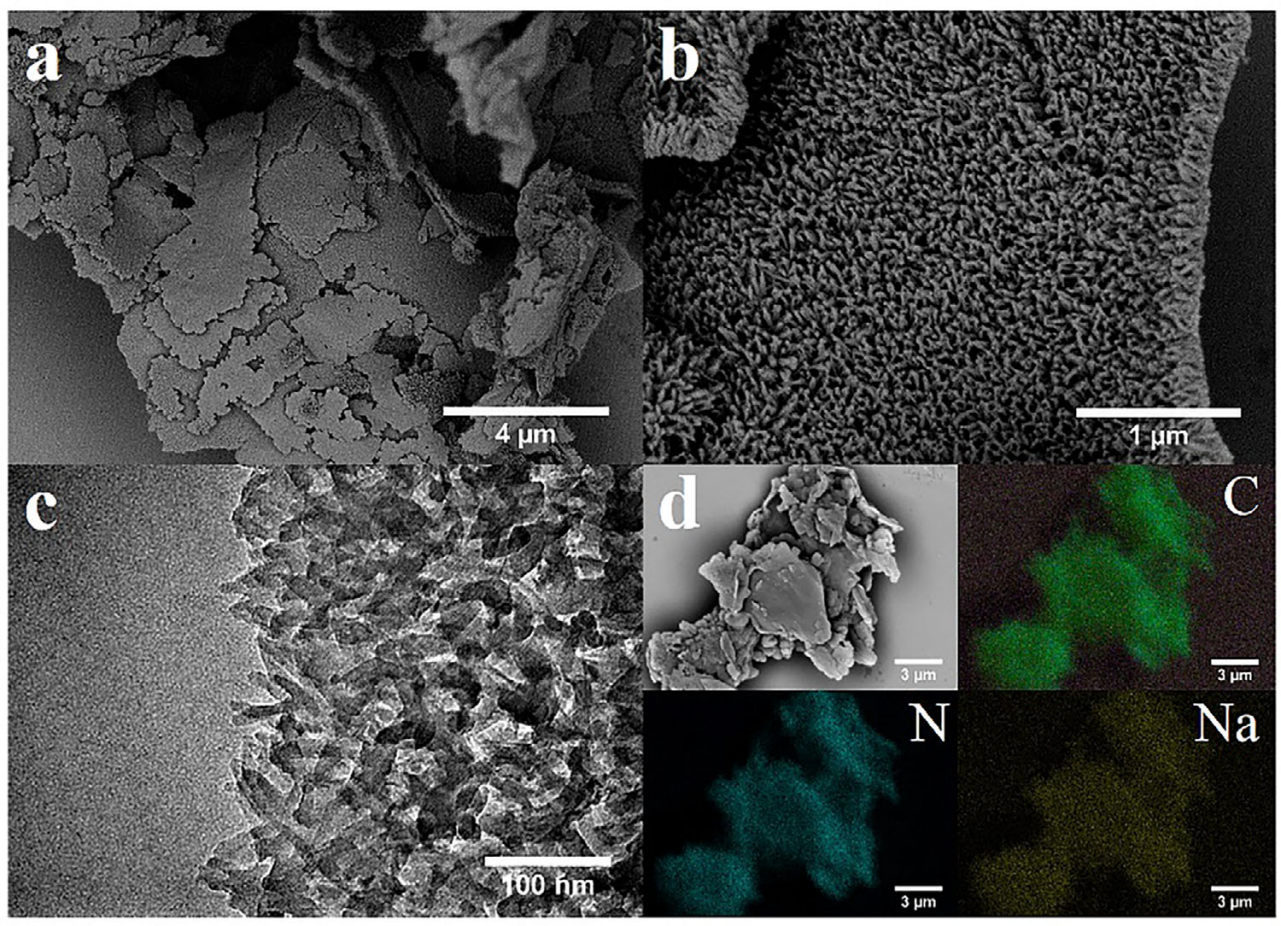
a,b) FESEM images of PH‐C_3_N_5_‐Na, c) TEM image of PH‐C_3_N_5_‐Na, d) EDX mapping of PH‐C_3_N_5_‐Na.

The microstructure of the as‐obtained PH‐C_3_N_5_‐Na was first examined by field‐emission scanning electron microscopy (FESEM) and transmission electron microscopy (TEM). As shown in Figure S1 (Supporting Information) and **Figure** **2**a–c, PM‐C_3_N_5_ exhibits a bulk structure, while PH‐C_3_N_5_‐Na displays a three‐dimensional superstructure stacked by nanorods, which is similar with widely reported results in the synthesis of PCN through molten salt‐mediated strategy.^[^ ^21^, ^26^, ^27^ ^]^ This distinct morphological transformation prompted us to investigate the specific surface area, a critical parameter influencing photocatalytic performance. The specific surface area of PH‐C_3_N_5_‐Na is calculated to be 21.55 cm^2^ g^−1^ (Figure S2, Supporting Information), which is 2.6 times folder than that of PM‐C_3_N_5_ (8.21 cm^2^ g^−1^).

To study the chemical composition of the synthesized PH‐C_3_N_5_‐Na, energy‐dispersive X‐ray spectroscopy (EDX) analysis and chemical elemental analysis (EA) were performed. The elemental mapping (Figure 2d) evidences the existence of C, N, and Na, which are all distributed uniformly throughout the material. The C/N atomic ratios were further quantified by EA. As summarized in Table S1 (Supporting Information), the C/N ratio of PM‐C_3_N_5_ is 0.647 which is lower than that of melon‐type PCN (BM‐C_3_N_4_, 0.672), suggesting that rich presence of N in the PM‐C_3_N_5_ owing to the incorporation of triazole units in PCN framework.^[^ ^22^, ^28^ ^]^ For PH‐C_3_N_5_‐Na, the C/N ratio increased slightly to 0.689, suggesting an enhanced degree of condensation and poly(heptazine imide)‐type PCN is formed.^[^ ^28^, ^29^ ^]^

**Figure 3:**
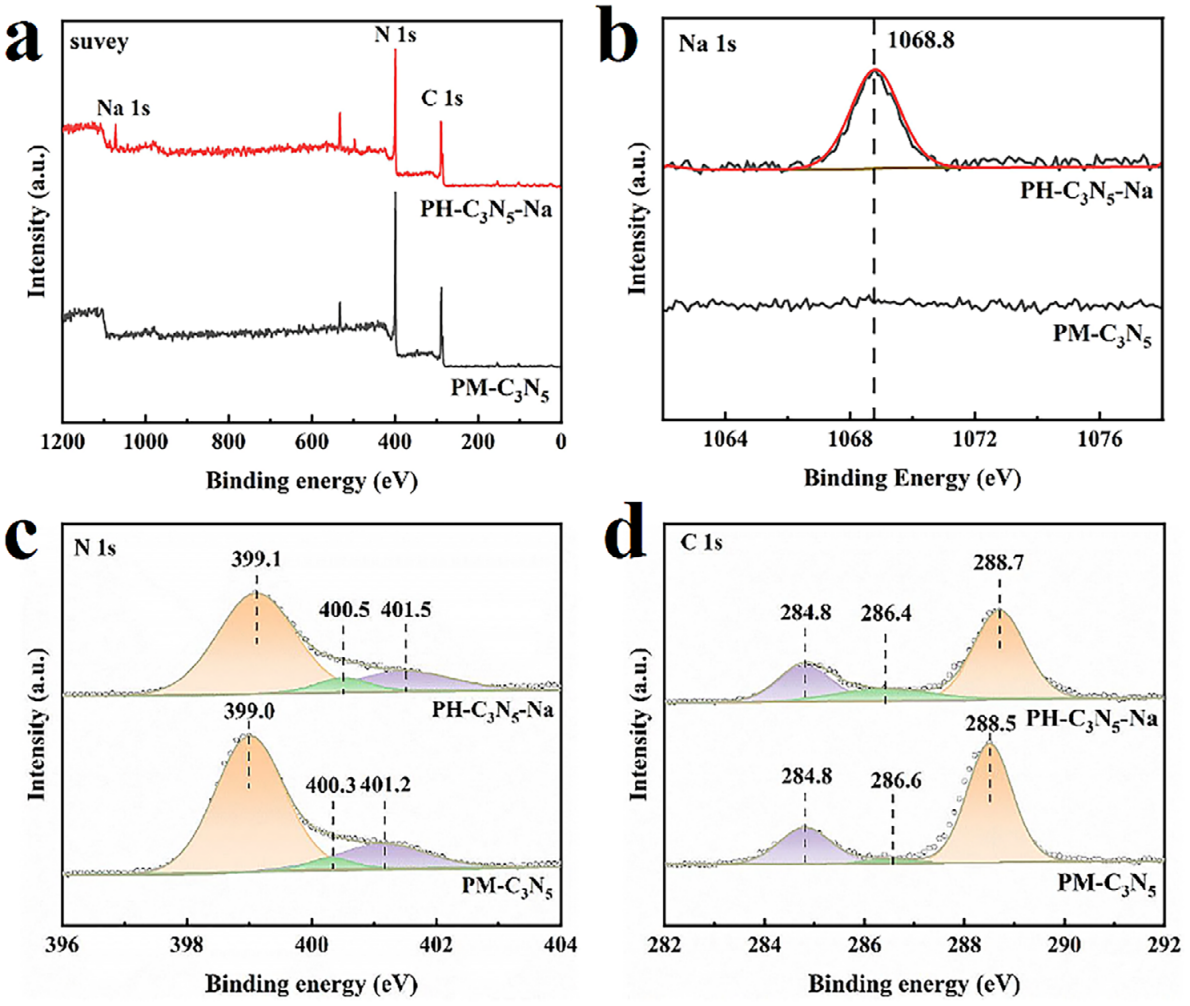
XPS survey spectra a) and high‐resolution XPS spectra in Na 1s b), N 1s c), and C 1s d) regions of PH‐C_3_N_5_‐Na and PM‐C_3_N_5_.

The elemental states in the samples were analyzed using X‐ray photoelectron spectroscopy (XPS). As shown in the XPS survey (**Figure** **3**a) and the high‐resolution Na spectra (Figure 3b), C, N, and Na are present in PH‐C_3_N_5_‐Na, whereas no Na was detected in PM‐C_3_N_5_. In addition, the absence of Li and Cl in the high‐resolution Li 1s and Cl 2p spectra (Figure S3, Supporting Information) further supports the hypothesis that Na has been successfully incorporated into the carbon nitride framework.^[^ ^21^ ^]^ This conclusion is further corroborated by complementary structural analyses obtained from Fourier‐transform infrared (FTIR) spectroscopy and solid‐state ^13^C nuclear magnetic resonance (^13^C NMR) spectroscopy. Regarding the C 1s spectra (Figure 3d), both samples exhibit three distinct peaks at ≈288, 286, and 284 eV. These peaks are attributed to sp^2^‐hybridized carbon in heptazine rings (N = C─N), sp^2^‐carbon bonded to terminal –NH_x_ groups (C–NH_x_), and adventitious hydrocarbons (C–C), respectively.^[^ ^29^, ^30^, ^31^ ^]^ In the same manner, the high‐resolution N 1s spectra (Figure 3c) reveal three distinct peaks at 398, 399.6, and 400.7 eV, corresponding to sp^2^‐hybridized nitrogen in heptazine (C = N–C), central nitrogen within the heptazine framework (N–(C)_3_), and terminal or bridged amino groups (C–N–H), respectively.^[^ ^29^, ^30^, ^31^ ^]^ Based on the above observations, it is reasonable to conclude that heptazine units serve as the primary structural motifs in the as‐prepared samples, as further corroborated by X‐ray diffraction (XRD) and FTIR analyses.

Further evidence for the formation of poly(heptazine imide)‐type PCN is provided by the XRD analysis (Figure 1b). For PM‐C_3_N_5_, the two dominant peaks at 12.9 and 27.6° originated from the (100) plane and (002) plane of polymeric melon‐type carbon nitride (PMCN).^[^ ^32^, ^33^, ^34^ ^]^

In the case of PH‐C_3_N_5_‐Na, two new peaks at 8.4 and 14.6°, which can be attributed to (100) and (110) planes of poly(heptazine imide) structure emerged, clearly indicating a transformation in the chemical configuration of the carbon nitride framework from melon‐type to heptazine‐imide‐type.^[^ ^21^, ^35^ ^]^ Additionally, the (002) diffraction peak shifts from 27.6 to 17.0° after molten‐salt‐assisted thermal condensation, indicating an increase in the interlayer spacing of PCN from 0.323 to 0.330 nm. This expansion can be reasonably ascribed to the incorporation of Na^+^ ions into the polymeric framework.^[^ ^21^, ^32^, ^35^ ^]^

To further reveal the functional groups and metalation of PH‐C_3_N_5_‐Na, FT‐IR and ^13^C NMR were measured and the results are shown in Figure 1c,d. All samples exhibit three evident vibration peaks for heptazine units, including the peak at 3100–3400, 1200–1650, and 810 cm^−1^, which belong to primary and secondary amines, aromatic carbon and nitrogen heterocycles, and the s‐triazine ring, respectively.^[^ ^36^ ^]^ Notably, the broad N–H stretching peak (3000–3400 cm^−1^) in PH‐C_3_N_5_‐Na slightly decreases and positively shifts toward a higher wavenumber (from 3100 to 3300 cm^−1^), suggesting the less amino group as a result of the molten‐salt induced deamination.^[^ ^37^ ^]^ This observation, in line with the XRD results, supports the structural transformation from melon‐type PCN with abundant –NH_2_ groups to a more condensed PHI‐type framework. Crucially, the PH‐C_3_N_5_‐Na shows two new absorption peaks at 990 and 1160 cm^−1^, which arises from the symmetric and asymmetric vibrations of C = N–C coordinated with alkali metal.^[^ ^30^, ^37^ ^]^ Further evidence of the incorporation of Na into PCN framework was further probed by solid‐state ^13^C NMR spectra (Figure 1d). Similar to PM‐C_3_N_5_, the NMR spectrum of PH‐C_3_N_5_‐Na showed two resolved resonances at ≈157 ppm and ≈164 ppm corresponding to the interior carbon atoms in the –N = CN_2_ (C(i)) and CN_2_(–NH_x_) (C(e)) moieties, respectively.^[^ ^27^, ^38^, ^39^ ^]^ This observation confirms that the heptazine‐based heterocyclic backbone is largely preserved after Na metalation. In comparison with the PCN, the peak (≈164 ppm), assigning to the C(e) atoms in CN_2_(−NH_x_), shows a 1.2 ppm shift for the PH‐C_3_N_5_‐Na_,_ likely due to Na incorporation into the heptazine skeleton, which increases the charge density of C(e) atoms.^[^ ^27^, ^39^ ^]^ Collectively, these results, in conjunction with the aforementioned structural analyses, clearly confirm that Na‐metalated N‐rich PHI was successfully synthesized via a molten salt‐mediated approach.

### 2.2 Electronic Properties and Band Structures

**Figure 4:**
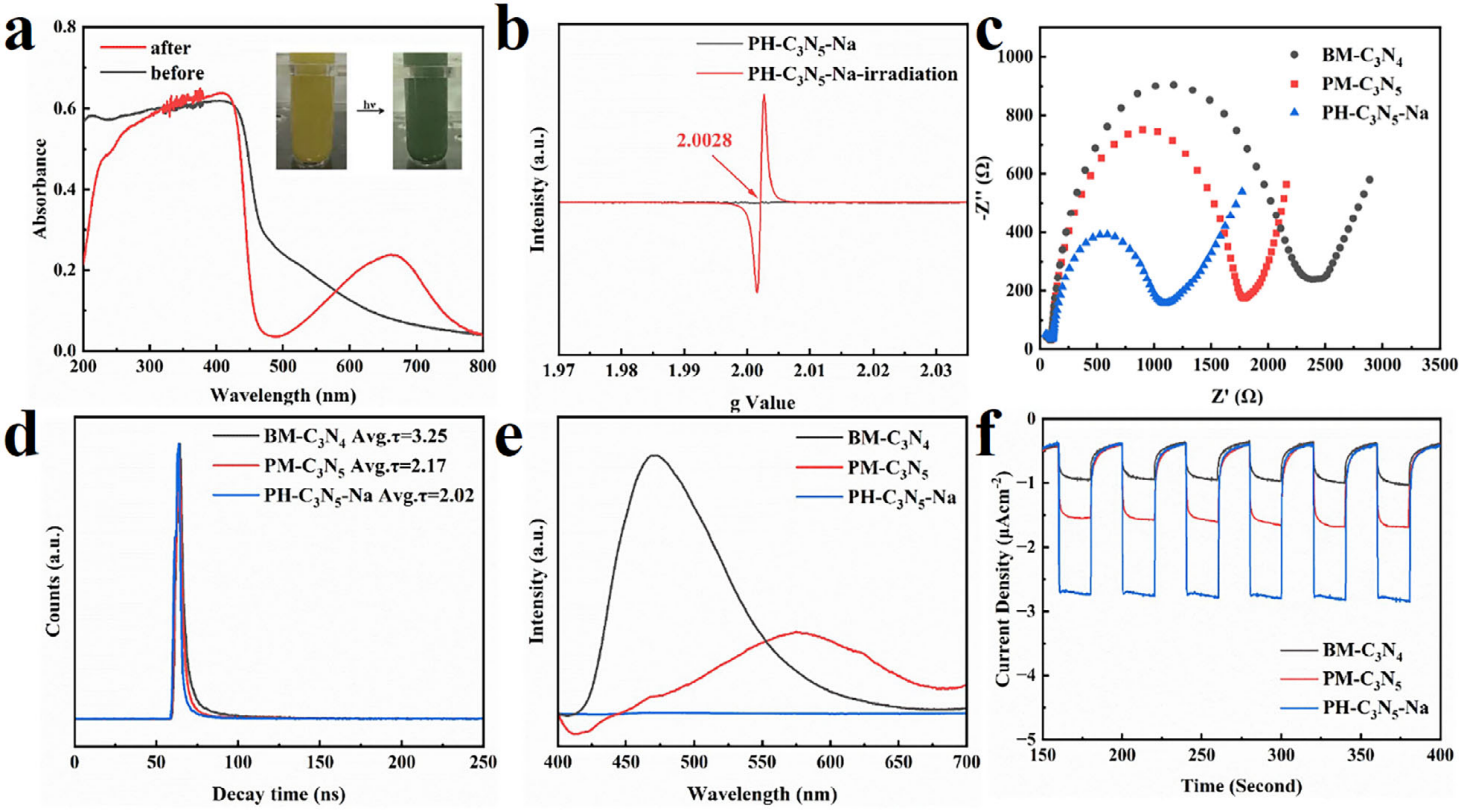
a) UV‐vis DRS of PH‐C_3_N_5_‐Na and the colloidal suspension of PH‐C_3_N_5_‐Na irradiated with visible light, b) EPR of PH‐C_3_N_5_‐Na and the colloidal suspension of PH‐C_3_N_5_‐Na irradiated with visible light, c) EIS Nyquist plots of BM‐C_3_N_4_, PM‐C_3_N_5_ and PH‐C_3_N_5_‐Na, d) time‐resolved transient PL spectra of BM‐C_3_N_4_, PM‐C_3_N_5_ and PH‐C_3_N_5_‐Na, e) steady‐state PL spectra of BM‐C_3_N_4_, PM‐C_3_N_5,_ and PH‐C_3_N_5_‐Na, f) transient photocurrent responses of BM‐C_3_N_4_, PM‐C_3_N_5,_ and PH‐C_3_N_5_‐Na.

Following the successful Na metalation of PCN, the in situ generation of solvated electrons by PH‐C_3_N_5_‐Na under light irradiation was systematically investigated. Upon exposure to visible light, the PH‐C_3_N_5_‐Na colloidal suspension underwent a noticeable color transition from yellow to green (**Figure** **4**a Inset), accompanied by the emergence of a broad absorption band spanning 460–750 nm (Figure 4a). The newly emerged absorption band unambiguously confirms the generation of solvated electrons on the surface of the photocatalyst.^[^ ^14^, ^21^, ^40^ ^]^ To gain further experimental support, electron paramagnetic resonance (EPR) spectroscopy was performed. As shown in Figure 4b, the unmodified PM‐C_3_N_5_ colloids displayed no discernible EPR signal, either in darkness or under light exposure, suggesting the absence of paramagnetic species. In marked contrast, light‐irradiated PH‐C_3_N_5_‐Na produced a distinct EPR resonance at a g value of 2.0028, a signature typically attributed to solvated electrons.^[^ ^20^, ^21^, ^40^ ^]^ These findings collectively demonstrate that Na metalation plays a pivotal role in enabling light‐driven generation of solvated electrons at the catalyst interface.

Beyond enabling the photogeneration of solvated electrons, Na metalation of PCN also exerts a pronounced impact on the kinetics of photoexcited charge carriers and optical absorption property. Electrochemical impedance spectroscopy (EIS) was employed to assess the electronic conductivity of the samples. Consistent with expectations, the arc radius (R) of PH‐C_3_N_5_‐Na is smaller than that of BM‐C_3_N_4_ and PM‐C_3_N_5_ (Figure 4c). The reduced R indicates that the metalation of PCN with Na significantly accelerates the transfer of photoexcited electrons, which is crucial for improving the separation of photoexcited charge carriers.^[^ ^37^, ^41^ ^]^ The fast transfer of photoexcited electrons is further verified by time‐resolved photoluminescence (TR‐PL) spectroscopy. The fitting PL decay data and average lifetime (τ_avg_) of these samples are shown in Figure 4d. Similarly with EIS results, the average lifetime PH‐C_3_N_5_‐Na is smaller than that of BM‐C_3_N_4_ and PM‐C_3_N_5_, indicating the rapid migration of photoexcited carriers due to the incorporation of Na in PCN.^[^ ^42^, ^43^, ^44^ ^]^ As a result, the recombination of photoexcited carriers is suppressed, which can be evidenced by PL analysis (Figure 4e). Notably, BM‐C_3_N_4_ displays the most intense PL signal, whereas PH‐C_3_N_5_‐Na shows markedly quenched emission. This pronounced PL suppression suggests that the synergistic effects of nitrogen enrichment and Na incorporation effectively inhibit the radiative recombination of photoexcited charge carriers. Such suppression is indicative of more efficient charge separation and transport, which is critical for enhancing the overall photocatalytic activity.

**Figure 5:**
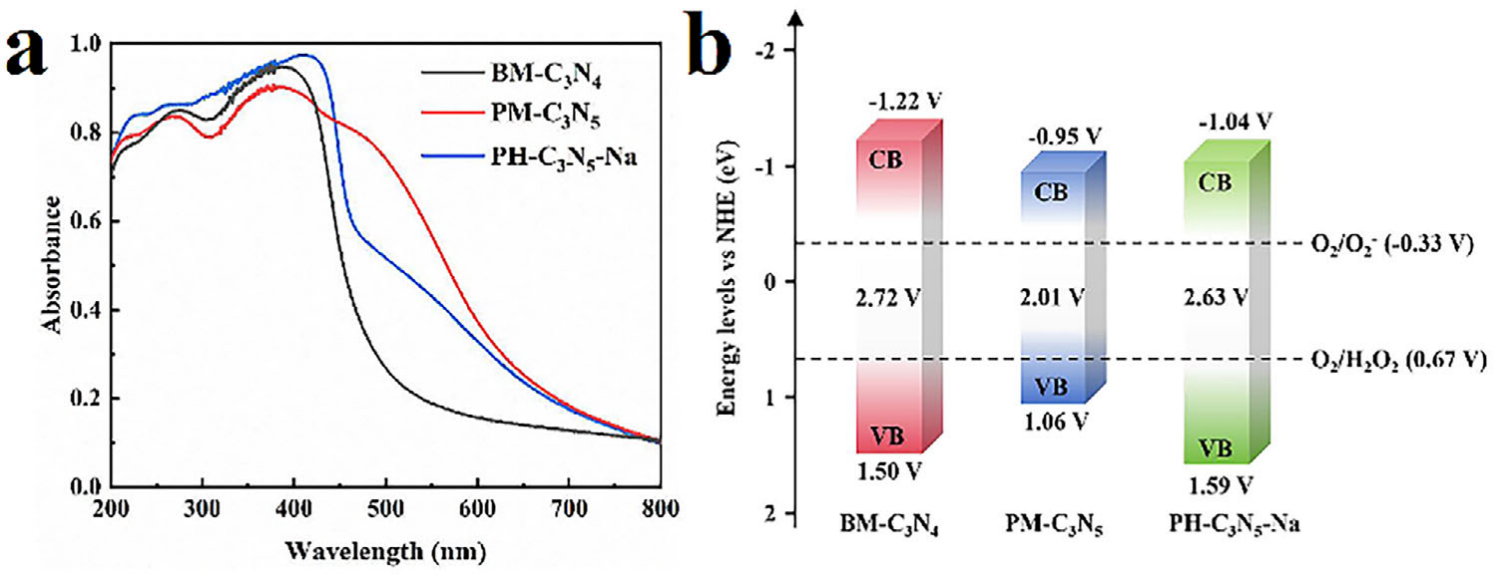
a) UV‐vis DRS of BM‐C_3_N_4_, PM‐C_3_N_5,_ and PH‐C_3_N_5_‐Na, b) schematic band structure of BM‐C_3_N_4_, PM‐C_3_N_5,_ and PH‐C_3_N_5_‐Na.

The influence of Na metalation on the optical absorption properties of the photocatalysts was investigated using UV‐vis diffuse reflectance spectroscopy (DRS). Compared to BM‐C_3_N_4_ (**Figure** **5**a), both PM‐C_3_N_5_ and PH‐C_3_N_5_‐Na display red‐shifted π → π^⁎^ absorption bands, along with the emergence of an additional n→ π^⁎^ transition in the range of 460‐700 nm.^[^ ^29^, ^41^, ^45^ ^]^ Interestingly, upon Na metalation, PH‐C_3_N_5_‐Na displays a distinct blue shift in the π→π^⁎^ transition, accompanied by a diminished intensity of the n→π^⁎^ transition. These spectral modifications suggest that Na metalation perturbs the electronic structure of the polymeric carbon nitride.

Correspondingly, Tauc plot analysis (Figure S5a, Supporting Information) reveals that the optical band gap of PH‐C_3_N_5_‐Na (2.63 eV) is 0.62 eV larger than that of pristine C_3_N_5_ (2.01 eV). This band gap broadening suggests that Na incorporation disturbs the π‐conjugated electronic structure of the carbon nitride framework. To further probe the electronic band structure, Mott‐Schottky measurements were conducted (Figure S5b, Supporting Information). The flat band potentials of PH‐C_3_N_5_‐Na, PM‐C_3_N_5_, and BM‐C_3_N_4_ are −1.42, −1.15, and −1.21 V (vs. Ag/AgCl), corresponding to conduction band (CB) potentials of −1.22, −0.95, and −1.04 V (vs. RHE), respectively. Combined with the band gap values, the valence band (VB) potentials were estimated, and the resulting band structures are illustrated in Figure 5b. It is worth noting that the deeper VB of PH‐C_3_N_5_‐Na implies the enhancement of oxidative power of photoexcited holes, thereby the photocatalytic two‐electron oxygen reduction pathway for H_2_O_2_ generation.^[^ ^29^, ^46^, ^47^ ^]^

### 2.3 Photocatalytic H_2_O_2_ Production

**Figure 6:**
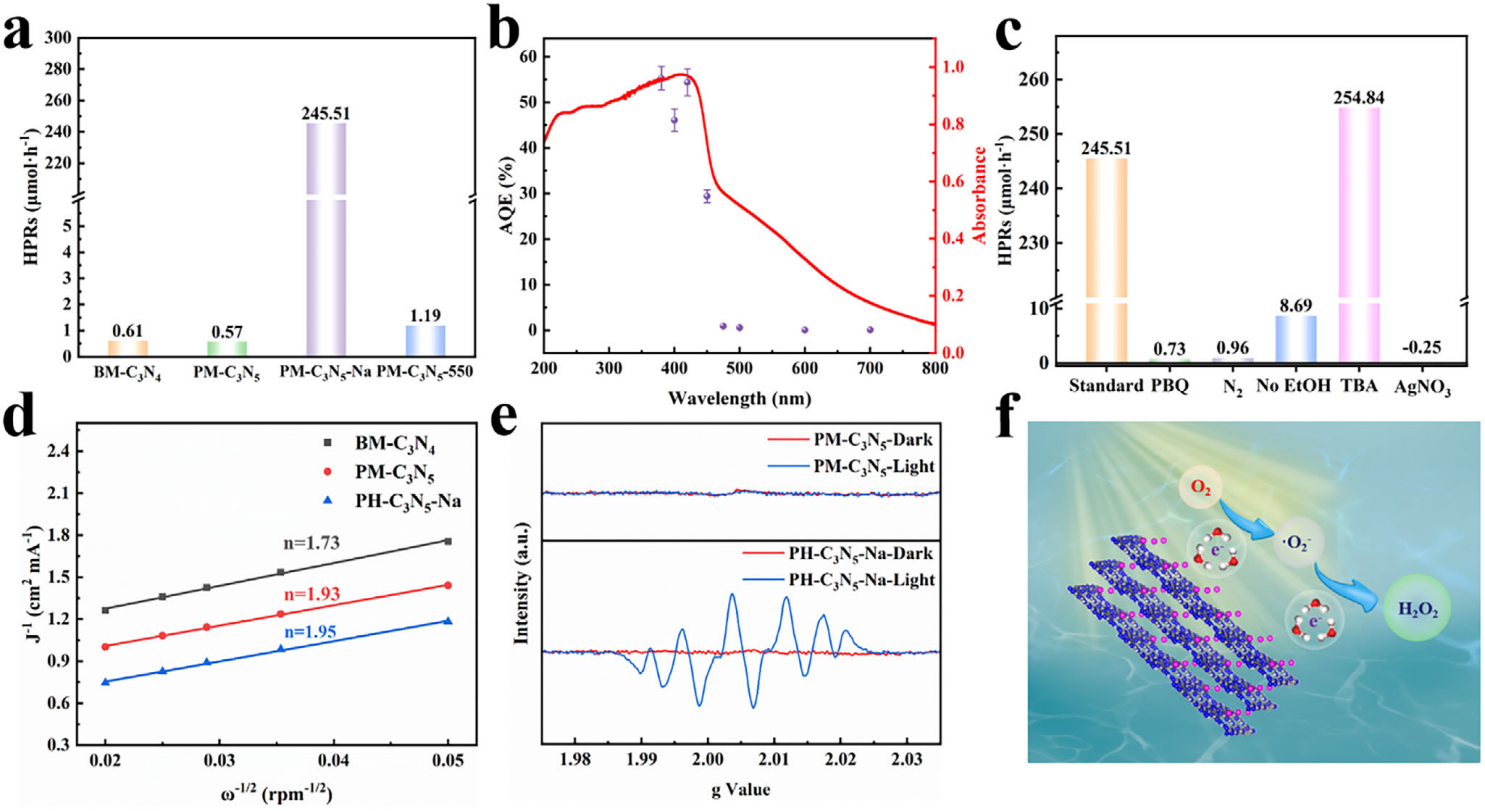
a) Photocatalytic H_2_O_2_ production rates of the as‐prepared samples under visible light (>420 nm) irradiation, b) wavelength dependent AQE of PH‐C_3_N_5_‐Na for H_2_O_2_ production, c) comparison of H_2_O_2_ production in 1 h under different conditions over PH‐C_3_N_5_‐Na, d) Koutecky‐Levich plots of the prepared samples (at 0.80 V vs. Ag/AgCl), e) EPR spectra of PM‐C_3_N_5_ and PH‐C_3_N_5_‐Na in the dark and under visible light, using DMPO as the spin‐trapping chemical, f) the plausible mechanism for photocatalytic reduction of O_2_ for H_2_O_2_ production over PH‐C_3_N_5_‐Na.

The photocatalytic reduction of O_2_ to H_2_O_2_ was performed under visible‐light irradiation (λ > 420 nm) using ethanol (EtOH) as a sacrificial electron donor. The hydrogen peroxide production rates (HPRs) of the synthesized carbon nitride‐based photocatalysts are compared in **Figure** **6**a. Both BM‐C_3_N_4_ and PM‐C_3_N_5_ exhibit negligible activity toward H_2_O_2_ generation, whereas Na modification results in a dramatic enhancement, with PH‐C_3_N_5_‐Na achieving a notably high production rate of 245.51 µmol·h^−1^. To clarify the contribution of Na metalation in boosting photocatalytic performance, PH‐C_3_N_5_‐Na was benchmarked against PM‐C_3_N_5_‐550 (Figure S7, Supporting Information), a control prepared under identical thermal conditions but without salt. Consistent with expectations, PH‐C_3_N_5_‐Na displays an H_2_O_2_ production rate 206 times greater than that of PM‐C_3_N_5_‐550.

Integrating the results from diffuse reflectance spectroscopy (DRS) and electron paramagnetic resonance (EPR) analyses, the pronounced activity enhancement is primarily attributed to the in situ formation of strongly reducing solvated electrons triggered by Na metalation. Additionally, the wavelength‐dependent apparent quantum efficiency (AQE) for H_2_O_2_ production over PH‐C_3_N_5_‐Na was systematically evaluated, with results depicted in Figure 6b. Impressively, PH‐C_3_N_5_‐Na achieves an AQE of 54.4% at 420 nm, surpassing most reported carbon nitride‐based photocatalysts (see Table S3, Supporting Information). A 4‐hour continuous visible‐light irradiation test confirmed the stable H_2_O_2_ production by PH‐C_3_N_5_‐Na (Figure S8, Supporting Information). Furthermore, the XRD, FTIR, FESEM and TEM characterizations (Figure S9, Supporting Information) reveal negligible structural changes post‐reaction, further evidencing the excellent photostability of PH‐C_3_N_5_‐Na.

### 2.4 Photocatalytic Mechanism for H_2_O_2_ Production

To elucidate the formation pathway for H_2_O_2_ generation, some control experiments were conducted first. It can be seen in Figure 6c that no detectable amount of H_2_O_2_ is generated as the O_2_ is replaced by N_2_.^[^ ^48^ ^]^ Moreover, the generation rate of H_2_O_2_ is suppressed by the presence of electron‐trapping agents, i.e., AgNO_3_. These results strongly demonstrate the formation of H_2_O is attributed to the reduction of O_2_ rather than the Oxidation of H_2_O.^[^ ^5^ ^]^

To investigate the number of transfer electrons in the ORR, the linear sweep voltammetry (LSV) curves (Figure S10, Supporting Information) were recorded using a rotating disk electrode (RDE) in an O_2_‐saturated phosphate buffer solution at different rotating speeds.^[^ ^4^, ^47^, ^49^ ^]^ Following Koutecky–Levich (K–L) equation (J^−1^ = J_L_^−1^ + J_K_^−1^ ), the corresponding K‐L plots(Figure 6d)were attained at constant electrode potentials (−1.0 V vs. Ag/AgCl). Derived from the slopes of K‐L curves with the equation (B = 0.2*nFν* ^−1/6^ *C*_0_ *D*_0_^2/3^), the electron transfer numbers (n) involved in the O_2_ reduction process on PM‐C_3_N_5_ and PH‐C_3_N_5_‐Na are determined to be 1.93 and 1.96, both of which are close to 2. These results confirm that the photocatalytic generation of H_2_O_2_ follows a two‐electron reduction mechanism.^[^ ^50^, ^51^, ^52^ ^]^

It is well established that photocatalytic H_2_O_2_ production via the 2e^−^ ORR can proceed through two distinct pathways: a direct one‐step 2e^−^ ORR and a stepwise 2e^−^ ORR pathway involving superoxide radicals (•O_2_^−^) as an intermediate. To identify the key intermediates involved in this process, radical scavenger experiments were conducted using p‐benzoquinone (PBQ) and tert‐butanol (TBA) as scavengers for •O_2_^−^ and hydroxyl radicals (•OH), respectively.^[^ ^5^, ^19^ ^]^ The addition of p‐benzoquinone (PBQ) as a •O_2_^−^ scavenger resulted in a sharp 99.7% decrease in the H_2_O_2_ production rate (Figure 6c), whereas the addition of TBA as a •OH scavenger had a negligible effect on H_2_O_2_ generation. These results indicate that •O_2_^−^ is the primary intermediate in the photocatalytic ORR over PH‐C_3_N_5_‐Na, while •OH does not play a significant role.

In addition, electron paramagnetic resonance (EPR) spectroscopy was employed to further corroborate this conclusion. Under visible‐light irradiation, a characteristic six‐line EPR signal corresponding to the DMPO–•O_2_^−^ species was observed for PH‐C_3_N_5_‐Na, whereas no such signal was detected in the absence of light (Figure 6e). In contrast, PM‐C_3_N_5_ failed to generate any detectable DMPO–•O_2_^−^ signal under either light or dark conditions, indicating that only the Na‐modified carbon nitride can photochemically produce superoxide radicals via solvated electrons.^[^ ^53^, ^54^ ^]^

Based on these results, it is clear that photocatalytic H_2_O_2_ generation over PH‐C_3_N_5_‐Na predominantly proceeds via a stepwise 2e^−^ ORR pathway, with •O_2_^−^ acting as the intermediate.^[^ ^55^, ^56^, ^57^ ^]^ Based on the above analyses, a plausible mechanism for photocatalytic O_2_ reduction to H_2_O_2_ over PH‐C_3_N_5_‐Na is proposed and illustrated in Figure 6f. Upon photoexcitation, electrons in the valence band (VB) of PH‐C_3_N_5_‐Na are excited to the conduction band (CB), generating photogenerated charge carriers (electrons and holes). The excited electrons are then transferred along the π‐conjugated framework to electron‐deficient Na sites on the surface of PH‐C_3_N_5_‐Na. These electrons rapidly become solvated in situ, forming solvated electrons, which exhibit enhanced stability and stronger reduction capability. Subsequently, the adsorbed O_2_ molecules undergo a single‐electron reduction to yield superoxide radical (•O_2_^−^). These reactive •O_2_^−^ species are then protonated and further reduced by solvated electrons, ultimately leading to the formation of H_2_O_2_. Meanwhile, the photogenerated holes are effectively scavenged by the oxidation of ethanol (EtOH), thereby suppressing charge recombination.

## 3 Conclusion

In summary, PH‐C_3_N_5_‐Na, a Na‐metalated and N‐rich poly(heptazine imide)‐type carbon nitride, was developed by employing 3‐amino‐1,2,4‐triazole as the precursor via a eutectic NaCl/LiCl molten salt‐assisted method. As anticipated, the resulting PH‐C_3_N_5_‐Na exhibits a substantially improved photocatalytic performance, achieving a H_2_O_2_ production rate of 245.51 µmol·h^−1^ and an unprecedented apparent quantum efficiency of 54.4% (420 nm), which is 206 times higher than that of PM‐C_3_N_5_‐550. The pronounced enhancement in photocatalytic H_2_O_2_ production is primarily attributed to the generation of surface‐bound solvated electrons on PH‐C_3_N_5_‐Na, which facilitates the stepwise formation of •O_2_^−^. Simultaneously, extending visible‐light absorption and enhancing the separation of photoexcited charge carriers, as induced by the incorporation of N‐rich units and Na into PCN, synergistically contribute to the markedly improved photocatalytic H_2_O_2_ production efficiency. This work highlights an effective strategy for enhancing photocatalytic H_2_O_2_ production via in situ generation of solvated electrons over alkali‐metalated polymeric carbon nitride.

## Conflict of Interest

The authors declare no conflict of interest.

## Supporting information



Supporting Information

## Data Availability

The data that support the findings of this study are available from the corresponding author upon reasonable request.
