# Supplementary material for: Solvated Electron‐Driven Stepwise ORR on Na‐Metalated N‐Rich Carbon Nitride for Efficient Photocatalytic H2O2 Production
Source: Adv Sci (Weinh). 2025 Oct 20;13(1):e16471. doi: 10.1002/advs.202516471 (PMC12767047; doi:10.1002/advs.202516471)
Supplement: Supplementary file 1 — Supporting Information [file ADVS-13-e16471-s001.docx]

Supporting Information

**Solvated Electron-Driven Stepwise ORR on Na-Metalated N-Rich Carbon Nitride for Efficient Photocatalytic H_2_O_2_ Production**

Jianan Feng, Weilin Qin, Li Shangguan, Hui Zhang, Jianhua Sun^*^, Shunping Sun, Yu Guo, Weiwei Lei^*^

## Experimental

### 1. Materials

3-Amino-1,2,4-triazole (3AT, 99%)， melamine (MA, 99%) and Hydrogen peroxide (H_2_O_2_, 30%) were purchased from J&K Scientific. Sodium chloride (NaCl), lithium chloride (LiCl), silver nitrate (AgNO_3_), isopropyl alcohol (IPA), *p*-benzoquinone (PBQ), tert-butanol (TBA), ethylene glycol (EG), triethanolamine (TEA), and absolute ethanol (EtOH) were all analytical grade and acquired from Sinopharm Chemical Reagent Co., China. Nafion (5%), 5,5-Dimethyl-1-pyrroline N-oxide (DMPO) was supplied by Sigma-Aldrich Chemical Reagent Co., Ltd. All chemicals were used as received without further purification. Ultrapure water was used throughout all experiments.

### 2. synthesis of samples

#### 2.1 Synthesis of PH-C_3_N_5_-Na

PH-C_3_N_5_-Na was synthesized via a two-step molten salt-assisted method. As a first step, 6.00 g of 3AT was placed in a 100 mL covered crucible and calcined in air at 500 °C for 4 h with a heating rate of 2.3 °C min^−1^. After natural cooling, the resulting reddish-brown solid was ground into a powder and named as PM-C_3_N_5_. After that, 1.00 g of PM-C_3_N_5_ was thoroughly ground with 10.0 g of NaCl/LiCl eutectic salt mixture (35/65 wt%). The mixture was transferred to a capped vessel and thermally treated at 550 °C for 4 h under the same heating rate. The resulting solid was thoroughly washed with boiling deionized water to remove residual salts and then dried under vacuum at 60 °C overnight. The final product was denoted as PH-C_3_N_5_-Na.

#### 2.2 Synthesis of PM-C_3_N_5_-550

PM-C_3_N_5_-550 was synthesized following the same procedure as PH-C_3_N_5_-Na, with the exception that the precursor was not ground in the NaCl/LiCl mixture.

#### 2.3 Synthesis of BM-C_3_N_4_

Bulk melon-based carbon nitride (BM-C_3_N_4_) was synthesized through a conventional thermal polycondensation approach. Specifically, 5.00 g of MA was placed in a 30 mL covered crucible and heated at 550 °C for 4 h in air with a heating rate of 2.3 °C min^−1^. After natural cooling to room temperature, the resulting light-yellow agglomerates were ground into a fine powder for further use.

### 3. Characterizations

Field-emission scanning electron microscopy (FESEM) was conducted using a NOVA NanoSEM 450 (FEI, Thermo Fisher Scientific) equipped with an Oxford EDS system for elemental analysis. Transmission electron microscopy (TEM) was performed on a JEM-2100 (JEOL) operated at 200 kV. Nitrogen adsorption-desorption isotherms were obtained using a Quadrasorb 2MP surface area and porosity analyzer (Quantachrome, USA) after degassing the samples at 150 °C under vacuum overnight. Elemental analysis (EA) was carried out on a Vario EL Cube (Elementar, Germany). X-ray diffraction (XRD) patterns were recorded using a PW3040/60 X’Pert PRO diffractometer (PANalytical) with monochromated Cu Kα radiation (λ = 0.15406 nm) at a scan rate of 0.2 ° s^–1^. Fourier-transform infrared (FT-IR) spectra were acquired on a Spectrum 3 spectrometer (PerkinElmer) equipped with an attenuated total reflectance (ATR) accessory, at a resolution of 4 cm^–1^ over the range of 4000-500 cm^–1^. X-ray photoelectron spectroscopy (XPS) measurements were performed using an ESCALAB Xi^+^ (Thermo Fisher Scientific) with monochromatic Al Kα radiation. UV-vis diffuse reflectance spectra (DRS) were collected on a Lambda 850+ spectrophotometer (PerkinElmer) equipped with an integrating sphere. Time-resolved photoluminescence (TR-PL) and steady-state photoluminescence (PL) spectra were recorded on a FluoroMax-4 spectrofluorometer (HORIBA). Electron paramagnetic resonance (EPR) measurements were conducted on an ESR A300 spectrometer (Bruker) at room temperature.

### 4. Photoelectrochemical measurements

Electrochemical and Transient photoelectrochemistry (TPC) measurements were carried on CHI760E workstation (Shanghai, China) by a conventional three-electrode system. GCE, ITO electrode and rotating disk electrode were modified as working electrodes, Ag/AgCl (saturated KCl) and platinum wire were served as reference and counter electrodes, respectively. The samples of modifying working electrode were prepared by dispersing 5 mg different power samples in 1 mL 0.02 % Nafion solution (V_water_:V _ethanol_ = 1:1), followed by ultrasonication for 30 min to form homogeneous slurry. Then, the slurry was uniformly drop-casted on different electrode surface with the loading of 357μg/cm^2^, and finally dried at room temperature.

Mott-Schottky plots were measured by the modified GCE in 0.2 M Na_2_SO_4_ solution, the potential window was from 0.2 to 1.0 V with the frequencies of 3000Hz, 3500Hz and 4000Hz. Electrochemical impedance spectroscopy (EIS) plots were carried out by the modified GCE in 0.1 M KCl containing 5.0 mM K_3_Fe(CN)_6_/K_4_Fe(CN)_6_ (1:1) solution, the frequency range from 0.1 Hz to 100 kHz with an AC amplitude of 10 mV. TPC curves were measured by the modified ITO electrode in 0.2 M Na_2_SO_4_ solution. A 300 W Xe arc lamp was selected as the visible light (> 420 nm) with an applied potential of -0.2 V and an interval of 20 s between switching the lamp on and off.

Linear sweep voltammetry (LSV) curves were performed by the modified rotating disk electrode (RDE-3A, BAS, Japan) for calculating the average transfer electron number (n) of O_2_ reduction. The electrolyte was 0.1 M KOH solution with O_2_-saturated, the potential window was from -1.0 to 0.2 V with the scan rate of 20 mV/s and the rotating rates of 400-2500 rpm. The average transfer electron number (n) was obtained by Koutecky-Levich (K-L) equation:

*J*^−1^ = *J*_k_^−1^ + B^−1^*ω*^−1/2^

B = 0.62*nFν*^−1/6^*C*_0_*D*_0_^2/3^

where *J*, *J*_k_ and ω are the measured, kinetic-limited and angular velocity of the rotating electrode (rpm), respectively. *F* and *ν* are the Faraday constant (96485 C mol^−1^) and kinetic viscosity of water (0.01 cm^2^ s^−1^), *C*_0_ and *D*_0_ are the bulk concentration of O_2_ in water (1.2 × 10^−3^ M) and the diffusion coefficient of O_2_ (1.9 × 10^−5^ cm^2^ s^−1^). From the slope *B* of the straight line, *j*^−1^ versus *ω*^−1/2^, the parameter n can be estimated.

### 5. Photocatalytic measurements

The photocatalytic reduction of O_2_ for H_2_O_2_ production was performed in a top-irradiation quartz reactor. Typically, 50 mg of the as-prepared photocatalyst was dispersed 50 mL ethanol aqueous solution (10 vol%). The suspension was stirred in the dark for 30 min under continuous O_2_ bubbling (0.5 L·min^–1^) to establish adsorption-desorption equilibrium. Subsequently, the resulting dispersion was irradiated using a 300 W Xe lamp equipped with a cut-off filter (λ > 420 nm), while the reaction temperature was maintained at 20 °C by a circulating water system. To monitor the photocatalytic production of H_2_O_2_, 0.5 mL aliquots were collected at the given time interval and filtered through Millipore filters (0.45 μm) to remove the photocatalyst.

The concentration of H_2_O_2_ was determined via a UV-vis spectrophotometric method based on the horseradish peroxidase (HRP)/3,3’,5,5’-tetramethylbenzidine (TMB) reaction system. Typically, 100 μL of the filtered reaction solution was mixed with 2 mL of Na_2_HPO_4_/NaH_2_PO_4_ buffer (0.1 M, pH 7.4), 10 μL of TMB solution (0.1 M), and 10 μL of HRP solution (0.1 M). After standing for 10 min at room temperature, the solution turned blue. Subsequently, 200 μL of H_2_SO_4_ (3 M) was added to quench the reaction, resulting in a yellow chromophore. The absorbance at 450 nm was measured using a UV-vis spectrophotometer, with appropriate dilution applied when necessary. The photocatalytic rate of H_2_O_2_ production was then calculated based on a standard calibration curve (Figure S6).

The apparent quantum efficiency (AQE) for H_2_O_2_ production was investigated with various band-pass filters and calculated using equation as follows:

$$\text{AQE =}\frac{N_{e}}{N_{p}}\text{=}\text{ }\frac{\text{2 ×n(}H_{2}O_{2}\text{)×}\text{N}\text{A}\text{ }\text{×}\text{h}\text{ × }\text{c}}{\text{ }\text{S }\text{×}\text{P }\text{×}\text{t}\text{ × }\text{λ}}\text{ × 100\%}$$

Where N_e_ is the amount of reaction electrons, N_p_ is the amount of incident photons, M is the amount of H_2_O_2_ molecules, N_A_ is Avogadro’s constant (6.022×10^23^ mol^−1^), h is the Planck constant (6.626×10^−34^ J·s), c is the speed of light (3.00×10^8^ m·s^−1^), S is the irradiation area (19.64 cm^2^ in this case), P is average irradiation intensity of the incident monochromatic light, t is the photoreaction time (3600 s in this case), and λ is the wavelength of the monochromatic light.

By integrating above formulas, the AQE is obtained as follows:

$$\text{AQE =}\frac{\text{ }\text{3.39}\text{×}{10}^{3}\text{ }\text{×}\text{n}\text{(}H_{2}O_{2}\text{)}}{\text{ }\text{P }\text{×}\text{λ}}\text{ × 100\%}$$

The average intensity of irradiation was measured by a Newport Oriel 91150 V reference cell. The measured values of *P* and *n*(H_2_) are listed in Table S2.


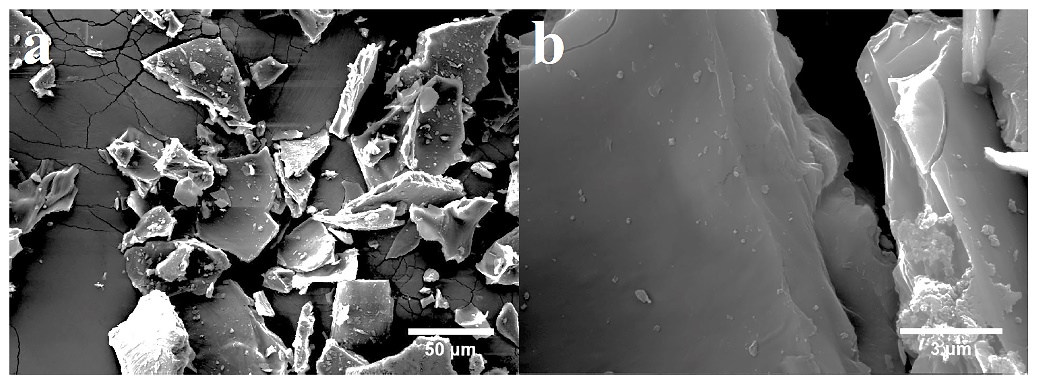


**Figure S1** FESEM of PM-C_3_N_5_.


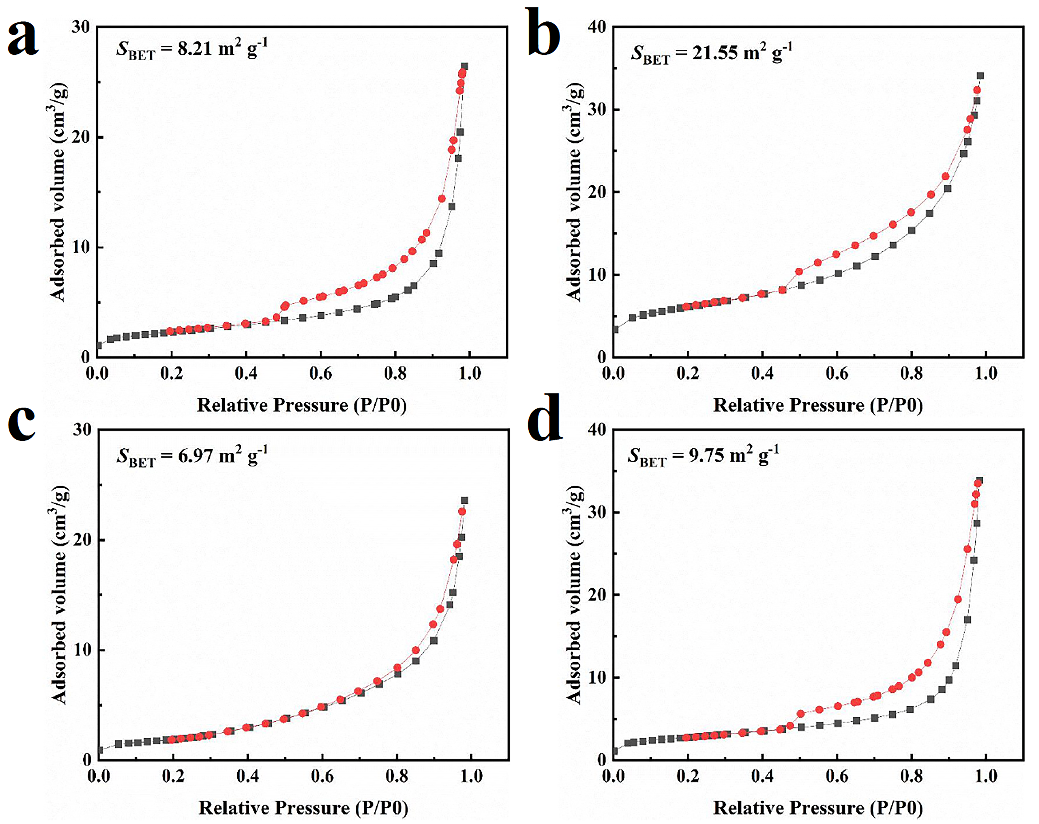


**Figure S2** N_2_ adsorption-desorption isotherm curves of the as-prepared PM-C_3_N_5_ (a), PM-C_3_N_5_-Na (b), BM-C_3_N_4_ (c), and PM-C_3_N_5_ (d).

**Table S1** Elemental compositions of the samples.

| Catalyst | C (wt%) | N (wt%) | H (wt%) | C/N molar ratio |
| --- | --- | --- | --- | --- |
| BM-C_3_N_4_ | 35.65 | 61.91 | 2.597 | 0.672 |
| PM-C_3_N_5_ | 33.92 | 61.17 | 2.172 | 0.647 |
| PH-C_3_N_5_-Na | 26.22 | 44.39 | 3.333 | 0.689 |
| PM-C_3_N_5_-550 | 34.43 | 61.88 | 1.92 | 0.649 |


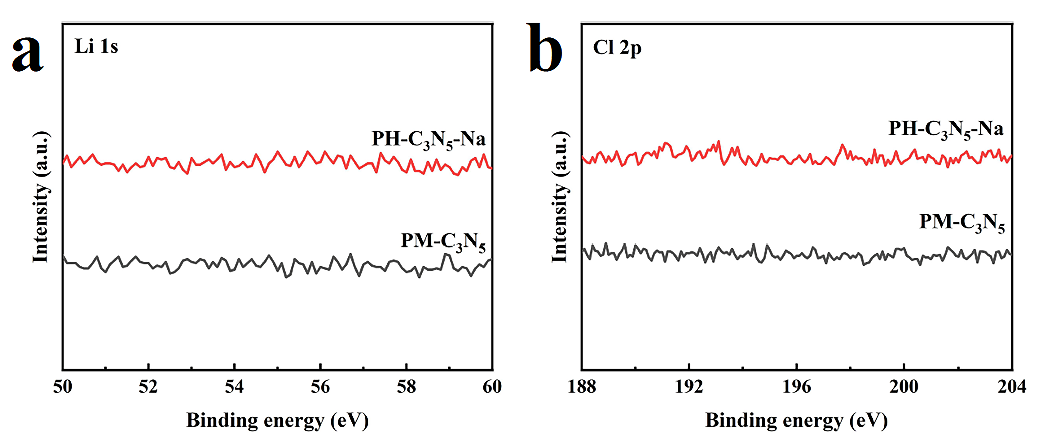


**Figure S3** High-resolution XPS spectra of PM-C_3_N_5_ and PH-C_3_N_5_-Na in Li 1s (a) and Cl 2p (b) regions.


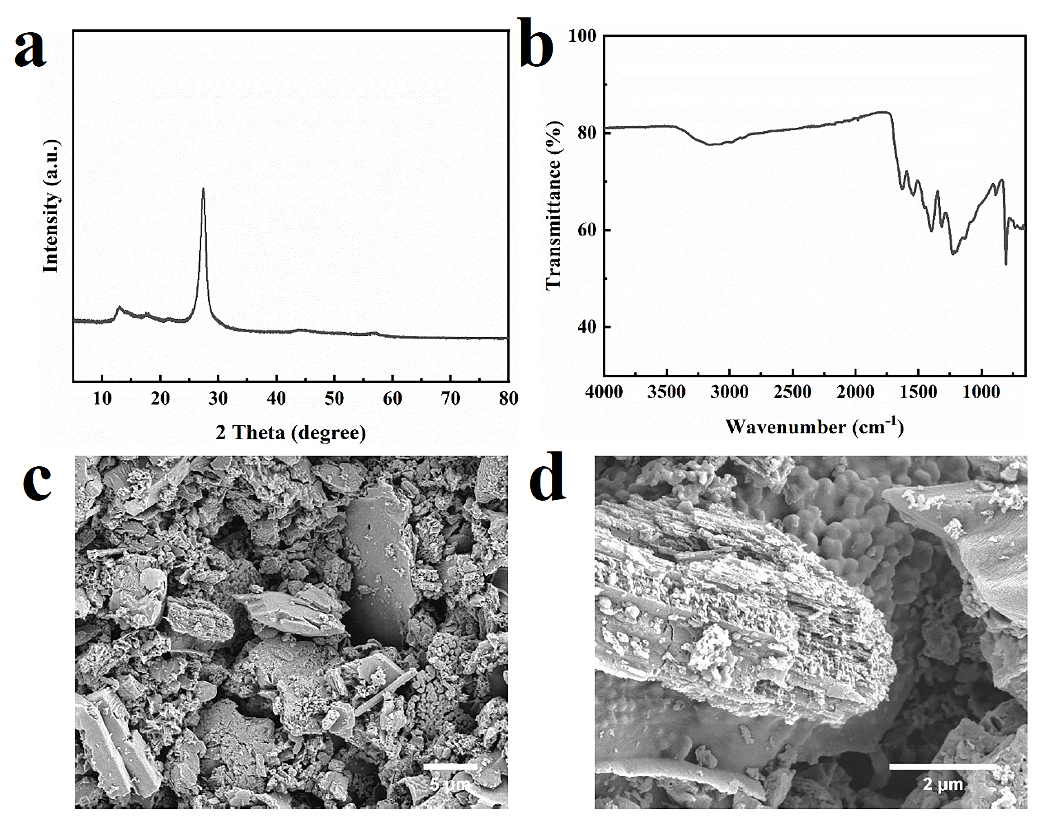


**Figure S4** XRD patterns (a), FTIR(b) spectra and FESEM images (c, d) of BM-C_3_N_4_.


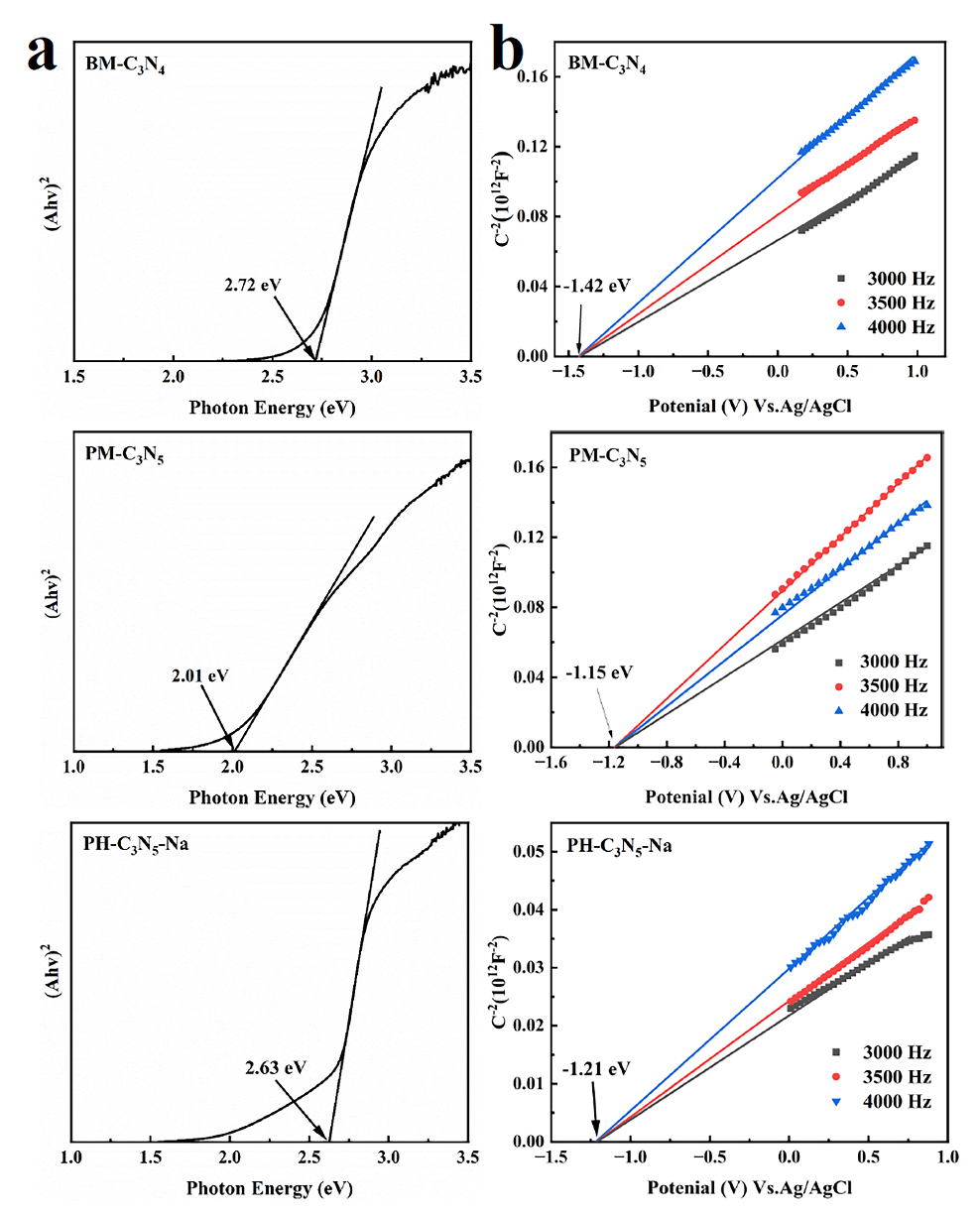


**Figure S5** Tauc plots (a) and Mott-Schottky plots (b) of BM-C_3_N_4_, PM-C_3_N_5_ and PH-C_3_N_5_-Na.


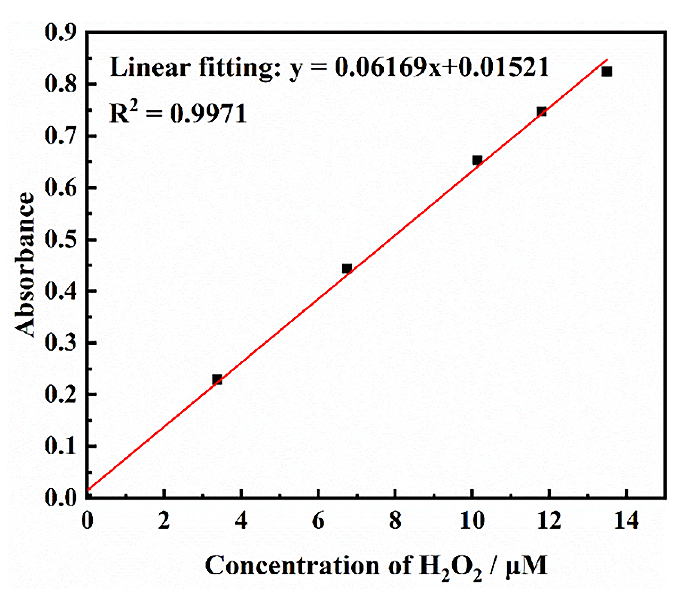


**Figure S6** The standard curves of absorption intensity at 450 nm vs. concentration of H_2_O_2_.


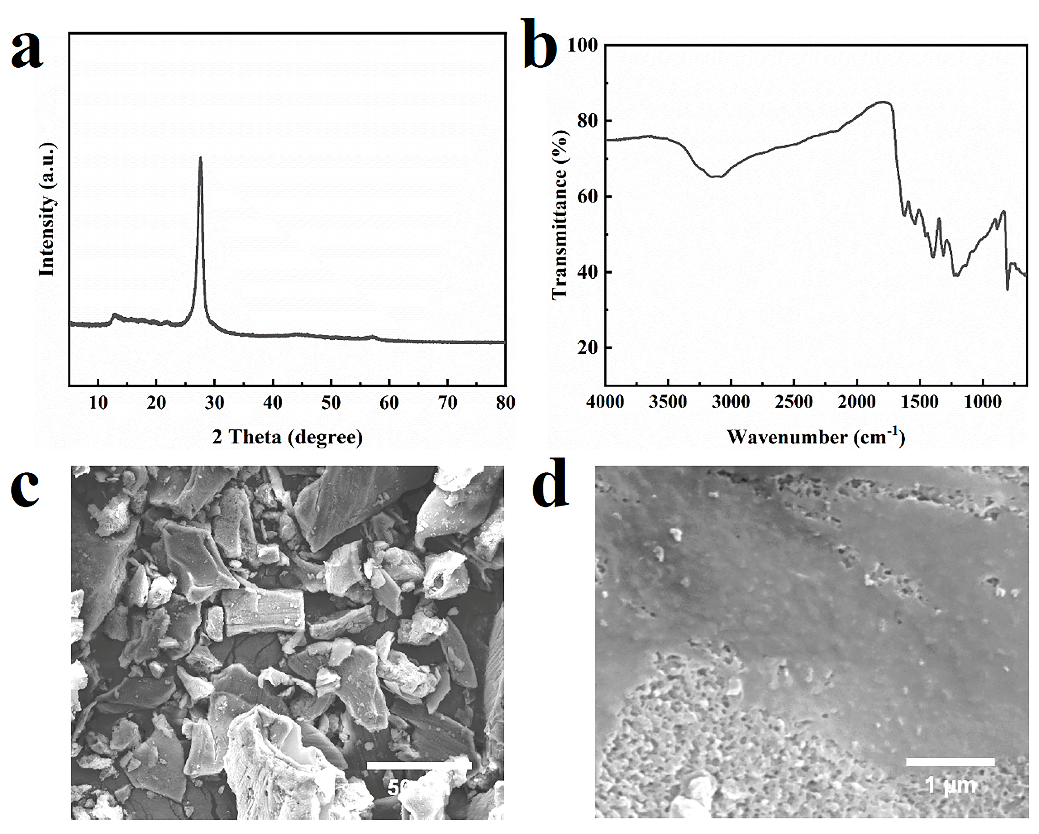


**Figure S7** XRD patterns (a), FTIR(b) spectra and FESEM images (c, d) of PM-C_3_N_5_-550.


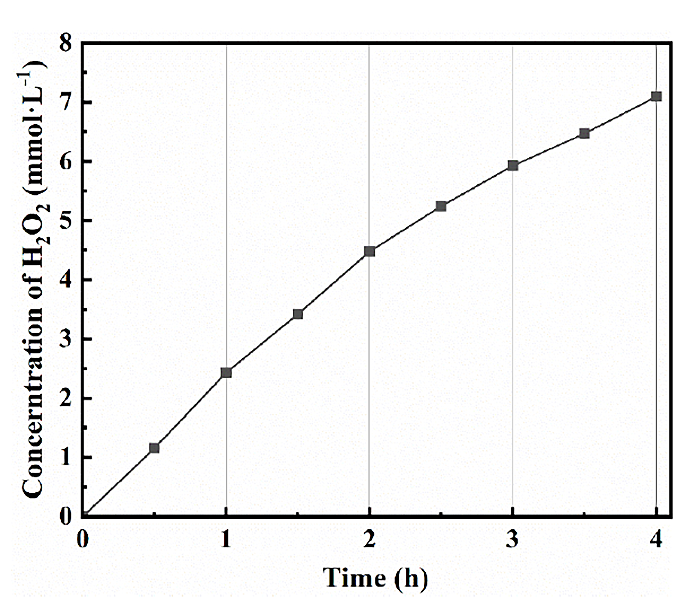


**Figure S8** The stability test of photocatalytic H_2_ production over PH-C_3_N_5_-Na.

**Table S2** The measured data and corresponding AQE of PH-C_3_N_5_-Na

| *λ* (nm) | P | *n*(H_2_O_2_) (μmol) | | | AQE (%) | | | |
| --- | --- | --- | --- | --- | --- | --- | --- | --- |
|  | (mW·cm^-2^) | 1 | 2 | 3 | 1 | 2 | 3 | Average |
| 380 | 1.863 | 109.7 | 116.6 | 120.2 | 52.5 | 55.8 | 57.5 | 55.3 |
| 400 | 3.420 | 196.5 | 185.3 | 176.8 | 48.7 | 45.9 | 43.8 | 46.1 |
| 420 | 1.535 | 102.3 | 109.6 | 98.53 | 53.7 | 57.6 | 51.8 | 54.4 |
| 450 | 2.548 | 103.1 | 94.21 | 101.3 | 30.5 | 27.8 | 29.9 | 29.4 |
| 475 | 3.500 | 4.362 | 4.475 | 4.579 | 0.889 | 0.912 | 0.933 | 0.911 |
| 500 | 1.955 | 1.783 | 1.719 | 1.631 | 0.618 | 0.596 | 0.565 | 0.593 |
| 600 | 5.590 | 0.401 | 0.432 | 0.390 | 0.040 | 0.044 | 0.039 | 0.041 |
| 700 | 5.270 | 0.9930 | 0.970 | 0.984 | 0.091 | 0.089 | 0.090 | 0.090 |

**Table S3** Comparison of AQE for H_2_O_2_ production at 420 nm between this work and previous studies.

| Photocatalyst | Dosage  (mg·mL^−1^) | | Sacrificial | AQE (%) | | Ref | |  |
| --- | --- | --- | --- | --- | --- | --- | --- | --- |
| PH-C_3_N_5_-Na | 0.5 | 10% ethanol | | | 54.4 | | This work | |
| CN-NH_4_-NaK | 0.5 | 10% isopropanol | | | 28.4 | | ^[1]^ | |
| Na-PCN | 1 | 10% ethanol | | | 22.3 | | ^[2]^ | |
| CN-KI_3_-KI-MV | 0.5 | 10% isopropanol | | | 27.56 | | ^[3]^ | |
| CN-2 | 0.5 | 10% ethanol | | | 48.8 | | ^[4]^ | |
| CC3N5 | 0.3 | 10% isopropanol | | | 12.86 | | ^[5]^ | |
| fl-CN-530 | 0.5 | 10% ethanol | | | 9 | | ^[6]^ | |
| ASCN-3 | 0.5 | 4-methoxybenzyl alcohol | | | 11.7 | | ^[7]^ | |
| CNR | 0.4 | 10% isopropanol | | | 14.58 | | ^[8]^ | |
| Nv-0.05 | 1 | 10% IPA | | | 29.3 | | ^[9]^ | |
| CHCN-0.02 | 1 | 10% ethanol | | | 28.1 | | ^[10]^ | |
| PCN-NaCA-2 | 0.3 | 3.5% glycerol | | | 11.8 | | ^[11]^ | |
| KCMCN | 0.4 | 0.5% isopropanol | | | 7.5 | | ^[12]^ | |
| ACNN | 0.5 | 10% isopropanol | | | 30.7 | | ^[13]^ | |
| AKMT | 0.5 | 10% ethanol | | | 100 | | ^[14]^ | |


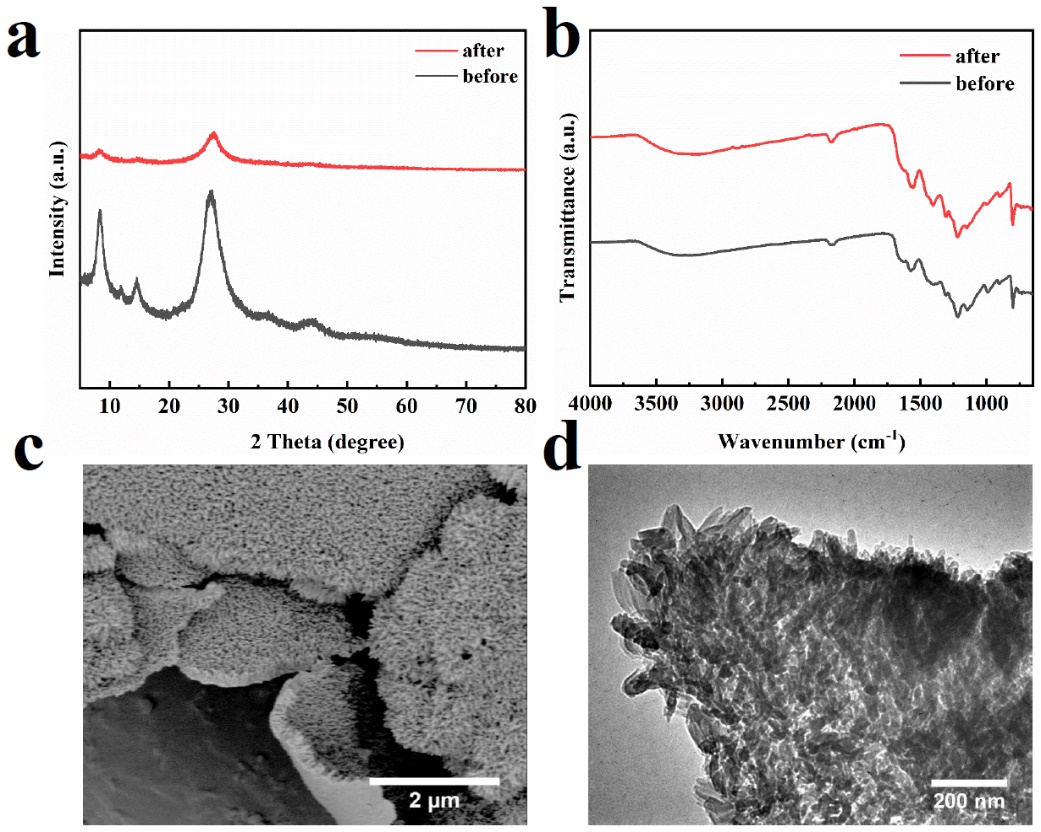


**Figure S9** XRD (a) and FT-IR (b), of PH-C_3_N_5_-Na before and after photocatalytic reaction, FESEM image (c) and TEM images (d) of PH-C_3_N_5_-Na after photocatalytic reaction.


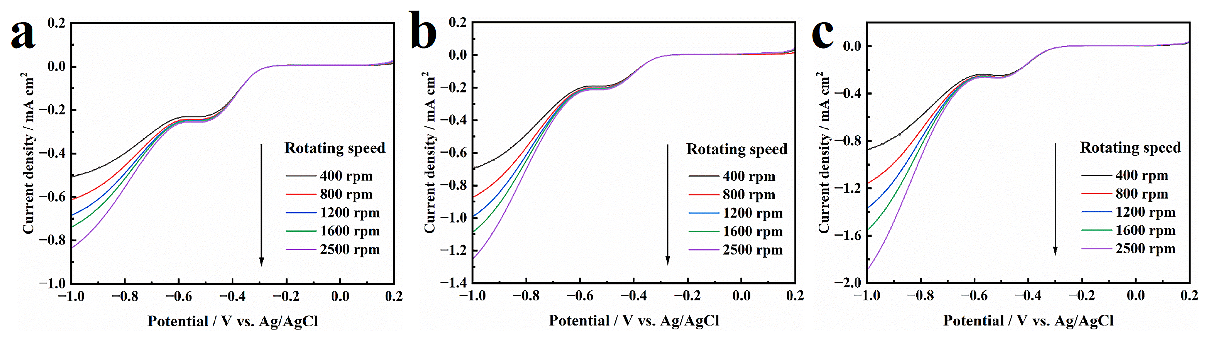


**Figure S10** LSV curves of BM-C_3_N_4_ (a), PM-C_3_N_5_ (b), and PH-C_3_N_5_-Na (c) measured on a RDE at different rotating speeds.

**References**

[1] F. He, Y. Lu, Y. Wu, S. Wang, Y. Zhang, P. Dong, Y. Wang, C. Zhao, S. Wang, J. Zhang, S. Wang, *Adv. Mater.* **2024**, 36, 2307490.

[2] L. Jian, Y. Dong, H. Zhao, C. Pan, G. Wang, Y. Zhu, *Applied Catalysis B-Environment and Energy* **2024**, 342, 123340.

[3] C.-W. Bai, L.-L. Liu, J.-J. Chen, F. Chen, Z.-Q. Zhang, Y.-J. Sun, X.-J. Chen, Q. Yang, H.-Q. Yu, *Nat. Commun.* **2024**, 15, 4718.

[4] Y. Wang, H. Zhang, D. Wei, S. Li, Y. Guo, J. Sun, *Chem. Commun.* **2024**, 60, 10732.

[5] S.-F. Ng, J. J. Foo, W.-J. Ong, *Materials Horizons* **2024**, 11, 408.

[6] B. Feng, Y. Liu, K. Wan, S. Zu, Y. Pei, X. Zhang, M. Qiao, H. Li, B. Zong, *Angew. Chem. Int. Ed.* **2024**, 63, e202401884.

[7] Q. Li, Y. Jiao, Y. Tang, J. Zhou, B. Wu, B. Jiang, H. Fu, *J. Am. Chem. Soc.* **2023**, 145, 20837.

[8] T. Yang, Y. Tang, F. Yang, J. Qu, X. Yang, Y. Cai, F. Du, C. M. Li, J. Hu, *Chem. Eng. J.* **2023**, 475, 146497.

[9] J. Li, J. Huang, G. Zeng, C. Zhang, H. Yu, Q. Wan, K. Yi, W. Zhang, H. Pang, S. Liu, S. Li, W. He, *Chem. Eng. J.* **2023**, 463, 142512.

[10] H. Luo, T. Shan, J. Zhou, L. Huang, L. Chen, R. Sa, Y. Yamauchi, J. You, Y. Asakura, Z. Yuan, H. Xiao, *Applied Catalysis B-Environment and Energy* **2023**, 337, 122933.

[11] Y. Zhao, P. Zhang, Z. Yang, L. Li, J. Gao, S. Chen, T. Xie, C. Diao, S. Xi, B. Xiao, C. Hu, W. Choi, *Nat. Commun.* **2021**, 12, 3701.

[12] Q. Cao, X. Wu, Y. Zhang, B. Yang, X. Ma, J. Song, J. Zhang, *J. Catal.* **2022**, 414, 64.

[13] S. Wu, H. Yu, S. Chen, X. Quan, *Acs Catalysis* **2020**, 10, 14380.

[14] P. Zhang, Y. Tong, Y. Liu, J. J. M. Vequizo, H. Sun, C. Yang, A. Yamakata, F. Fan, W. Lin, X. Wang, W. Choi, *Angew. Chem. Int. Ed.* **2020**, 59, 16209.
